# Small Intestinal Bacterial Overgrowth in Children with Short Bowel Syndrome

**DOI:** 10.3390/children12111550

**Published:** 2025-11-16

**Authors:** Hannah DeGonza, Thu Anh Pham, Rasha Elmaoued, Razan Alkhouri, Ricardo Orlando Castillo, Rajmohan Dharmaraj

**Affiliations:** Division of Gastroenterology, Department of Pediatrics, University of New Mexico, Albuquerque, NM 87131, USA; hdegonza@salud.unm.edu (H.D.); thapham@salud.unm.edu (T.A.P.); raelmaoued@salud.unm.edu (R.E.); ralkhouri@salud.unm.edu (R.A.); ricastillo@salud.unm.edu (R.O.C.)

**Keywords:** small intestinal bacterial overgrowth, short bowel syndrome, children, evaluation, management

## Abstract

Small Intestinal Bacterial Overgrowth (SIBO) is characterized by an abnormal proliferation of bacteria in the small intestine, leading to gastrointestinal symptoms and nutritional deficiencies. Short Bowel Syndrome (SBS), resulting from extensive surgical resection of the small intestine, predisposes children to SIBO due to anatomical disruptions, motility dysfunction, parenteral nutrition dependence, and immune dysregulation. Clinical manifestations of SIBO in SBS include bloating, diarrhea, malabsorption, and failure to thrive, with severe cases leading to complications such as D-lactic acidosis. Diagnosis remains challenging, with breath testing being the most commonly used method despite limitations in accuracy, especially in SBS patients. Jejunal aspiration, the gold standard, presents limitations due to contamination risks, potential for sampling error, and a relatively low diagnostic yield. Management involves antibiotics like rifaximin and metronidazole, alongside strategies to address anatomical dysfunction, optimize nutrition, and prevent recurrence. Adjunctive therapies, including probiotics and dietary modifications, show promise but require further validation in children. Emerging treatments, such as glucagon-like peptide-2 (GLP-2) analogs, may enhance mucosal integrity and reduce SIBO risk. Given the chronic and recurrent nature of SIBO in SBS, a multidisciplinary approach is essential, integrating gastroenterological, surgical, and nutritional care to effectively manage the condition. Future research should focus on improving diagnostic methods, refining treatment protocols, and exploring targeted therapies to enhance outcomes and quality of life for affected children.

## 1. Introduction

Small Intestinal Bacterial Overgrowth (SIBO) is defined as the pathological proliferation of bacteria within the small intestine, where microbial populations are typically sparse and tightly regulated by gastric acidity, intestinal motility, bile salts, and mucosal immunity. Disruption of these protective mechanisms leads to microbial dysbiosis, fermentation of luminal substrates, and subsequent gastrointestinal and nutritional complications. Although the gastrointestinal tract harbors a complex microbial ecosystem, the small bowel normally maintains bacterial counts below 10^3^ colony-forming units (CFUs) per milliliter. When this balance is disturbed, bacterial overgrowth can lead to malabsorption, mucosal inflammation, and systemic sequelae ranging from micronutrient deficiencies to metabolic derangements such as D-lactic acidosis [1,2,3].

In pediatric populations, SIBO has emerged as a frequent but underdiagnosed condition, particularly in children with underlying anatomic or functional intestinal disorders. Short Bowel Syndrome (SBS), resulting from extensive surgical resection of the small intestine, represents one of the most significant risk factors for SIBO. The incidence of SBS in the pediatric population has increased over the past two decades due to improved survival of premature infants and advances in neonatal surgical care [4,5]. Children with SBS experience profound alterations in intestinal anatomy, including the loss of the ileocecal valve, blind loop formation, and reduced mucosal surface area, each of which predisposes to bacterial stasis and retrograde colonization of the small bowel [6,7]. Furthermore, impaired motility, dependence on parenteral nutrition (PN), and immune dysregulation further compound the risk of SIBO, creating a cyclical relationship between bacterial overgrowth, mucosal injury, and intestinal failure [8,9,10].

Clinically, SIBO in children with SBS can be challenging to recognize because its manifestations—bloating, diarrhea, abdominal distension, and growth failure—often overlap with baseline symptoms of intestinal dysfunction. Persistent bacterial overgrowth may exacerbate nutrient malabsorption, interfere with intestinal adaptation, and delay the transition to enteral autonomy. A recent multicenter cohort from North America reported that up to 70% of children with SBS on long-term PN developed at least one confirmed or suspected episode of SIBO, underscoring its clinical importance [11]. Diagnostic methods remain imperfect: breath tests offer limited sensitivity in altered anatomy, while jejunal aspirates, though definitive, are invasive and prone to sampling error [12,13]. Recent advances in metagenomic sequencing have identified microbial signatures specific to SBS and SIBO, suggesting opportunities for molecular diagnostics and microbiome-targeted therapy [7,10]. Novel interventions—glucagon-like peptide-2 (GLP-2) analogs, probiotics, and dietary strategies—show potential to restore microbial balance and improve intestinal function [14,15,16]. However, pediatric data remain limited, emphasizing the need for standardized diagnostic and therapeutic protocols.

This narrative review summarizes current evidence on the pathophysiology, prevalence, clinical manifestations, diagnostic evaluation, and management of SIBO in children with SBS. By integrating recent developments in microbiome science, diagnostic methods, and intestinal rehabilitation, this review aims to enhance understanding of the complex interplay between bacterial overgrowth and SBS, guiding clinicians toward evidence-based strategies that optimize outcomes and quality of life for affected children.

## 2. Pathophysiology

### 2.1. Small Intestinal Microbiota

**Figure 1:**
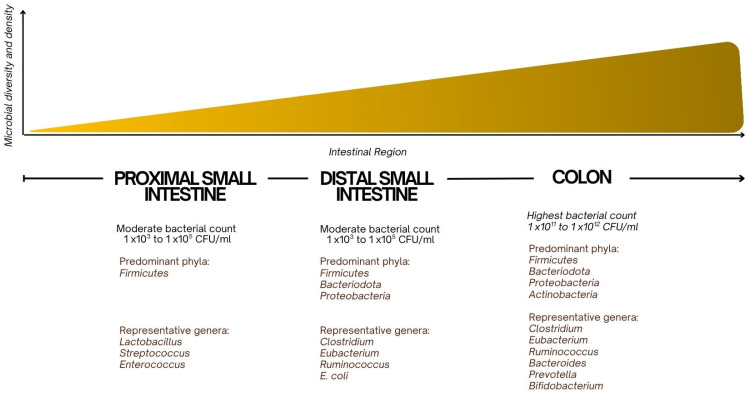
Bacterial Composition and Content of the Gastrointestinal Tract.

The small intestinal microbiota consists primarily of a variety of bacterial species working in homeostasis, with the composition of the microbiota shifting along the length of the intestine [3] (Figure 1). In the proximal small intestine, the microbiota is sparser and consists of Gram-positive bacteria, such as Lactobacillus and Streptococcus species, as well as some Enterococcus species [1]. These bacteria play a role in fermenting carbohydrates and preventing the overgrowth of harmful pathogens by maintaining an acidic pH. Lactobacillus species are known for producing lactic acid, which helps to lower the pH and create an environment hostile to pathogenic organisms [17].

In the distal small intestine, where bacterial density increases, *Bacteroides*, *Firmicutes,* and *Enterobacteriaceae* are more prevalent [8]. *Bacteroides* are involved in the breakdown of complex carbohydrates and are essential for the fermentation of fibers and other indigestible carbohydrates, producing short-chain fatty acids (SCFAs) such as butyrate, which provide energy to the epithelial cells of the intestinal lining [18]. *Firmicutes*, including *Clostridium* species, contribute to the breakdown of proteins and help regulate immune responses by promoting the development of regulatory T cells (Tregs) and maintaining intestinal homeostasis [18].

Additionally, the *Escherichia coli* group within *Enterobacteriaceae* can be both beneficial and pathogenic depending on the species and context. While some strains aid in the synthesis of essential nutrients, others, when overgrown, can lead to inflammation and disrupt the gut barrier [19].

### 2.2. Definition of SIBO

SIBO occurs when protective mechanisms that regulate microbial content in the small bowel are compromised, resulting in bacterial proliferation ≥ 10^3^ CFU/mL of luminal fluid from the proximal jejunum, as determined by quantitative aerobic and anaerobic bacteria cultures [1,3,6]. Importantly, it is not merely the quantity but the quality of the microbial overgrowth that contributes to pathogenesis. Anaerobes such as *Bacteroides* and facultative anaerobes like *Escherichia coli*, which are prevalent in the colon, colonize the small bowel and lead to fermentation of carbohydrates, deconjugation of bile salts, and direct mucosal injury [6]. The metabolic byproducts of these processes—particularly hydrogen and methane gases—contribute to abdominal distension, bloating, and altered bowel habits [3].

### 2.3. Risk Factors in Pediatric SBS

**Figure 2:**
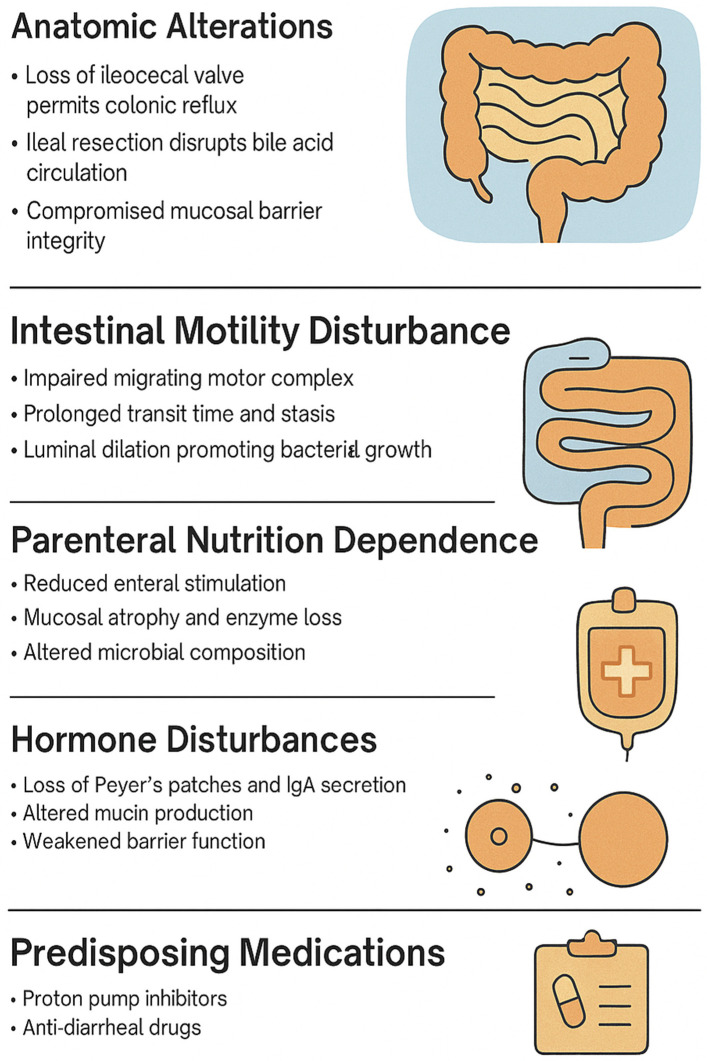
Pathophysiological mechanisms involved in the occurrence of Small Intestinal Bacterial Overgrowth in Pediatric Short Bowel Syndrome.

Pediatric SBS increases SIBO risk due to several risk factors, including anatomical abnormalities, motility dysfunction, reliance on parenteral nutrition, hormone disturbances, immune dysregulation, and predisposing medications (Table 1). These factors disrupt the gut microbiota, compromise mucosal barriers, and alter gastrointestinal motility, nutrient absorption, and immune function, thereby facilitating bacterial overgrowth (Figure 2).

#### 2.3.1. Anatomic Abnormalities

In pediatric populations with SBS, the risk of SIBO is markedly elevated due to a unique combination of anatomical and physiological disturbances. Because SBS is characterized by the surgical resection of extensive portions of the small intestine, the extent and site of the resection directly impact both nutrient assimilation and the likelihood of bacterial overgrowth [7]. Notably, the absence of the ileocecal valve, which is common in children with SBS, eliminates a crucial barrier preventing the retrograde migration of colonic microbiota into the small intestine [3,20]. In a retrospective analysis, 61.5% of children with SBS had an absent ileocecal valve [11].

Furthermore, resection of the terminal ileum—another frequent consequence of SBS—disrupts enterohepatic circulation and impairs the reabsorption of bile acids. As a result, bile salt malabsorption leads to steatorrhea, and deconjugated bile acids exert a direct cytotoxic effect on enterocytes, compounding mucosal damage [21]. These alterations, in turn, compromise the mucosal barrier and increase permeability, facilitating bacterial adherence and translocation. In animal models, loss of bile acid-mediated antimicrobial activity has been shown to significantly alter intestinal microbial composition, favoring the growth of opportunistic pathogens [22].

#### 2.3.2. Motility Dysfunction

Motility dysfunction is another central element in the pathogenesis of SIBO in children, particularly those with SBS. After bowel resection, the remaining intestine undergoes adaptive dilation and lengthening; however, this compensatory response is often associated with disordered motility and prolonged bowel transit times [20]. The migrating motor complex (MMC), which serves as a housekeeper by propelling luminal contents distally during fasting periods, is frequently impaired [1]. Stasis of intestinal contents permits bacterial colonization and the establishment of polymicrobial communities capable of altering nutrient metabolism and immune signaling [8,23]. Importantly, these bacterial communities do not merely persist passively but actively modify their environment by producing enzymes that can degrade disaccharides and oligosaccharides, increasing fermentative gas production [8].

#### 2.3.3. Parenteral Nutrition and Mucosal Adaptation

In children with SBS, long-term dependence on parenteral nutrition (PN) is another factor that contributes to microbial dysregulation in addition to overall disease burden [24,25]. The relative lack of enteral feeding leads to mucosal atrophy, decreased brush border enzyme activity, and reduced stimulation of gut-associated lymphoid tissue (GALT) [3,6]. Long-term reliance on PN disrupts the natural development and balance of intestinal microbiota, evidenced by altered microbial profiles in affected patients compared to those who received enteral feeds [7,26].

#### 2.3.4. Hormone Disturbances

Another significant factor in the pathophysiology of SIBO in SBS is the dysregulation of incretin hormones, particularly glucagon-like peptide-1 (GLP-1) and GLP-2 produced by L-cells. GLP-2 has trophic effects on the intestinal epithelium, contributing to mucosal integrity and nutrient absorption, while GLP-1 modulates motility and satiety [14]. After a massive bowel resection, the production of these hormones is impaired, particularly if the distal ileum, where L-cells reside, is removed. This hormonal deficiency contributes to delayed intestinal adaptation, impaired barrier function, and suboptimal motility, which, in combination, promote SIBO [11].

#### 2.3.5. Immune Dysregulation

Immune dysfunction also plays a prominent role in pediatric SIBO. In SBS, the loss of Peyer’s patches and M cells, particularly in the terminal ileum, results in impaired mucosal surveillance and reduced secretion of IgA, a key regulator of microbial composition [11,23]. Increased inflammation has also been linked to alterations in gene expression regulating mucous production and inability to trap bacteria intraluminally [8,26]. This immunologic insufficiency permits uncontrolled bacterial growth and facilitates systemic immune activation, increasing the risk of complications such as bacteremia, hepatic dysfunction, and chronic intestinal inflammation [6,27]. Chronic inflammation and associated liver disease exacerbate intestinal dysbiosis and bacterial translocation. Hepatobiliary dysfunction impairs bile acid circulation and antimicrobial activity, while systemic inflammation alters gut permeability and mucosal immunity, perpetuating the SIBO–liver injury cycle. Recognition of this gut-liver axis is essential in the management of SBS-related SIBO. Patients with SIBO exhibit a persistent depletion of beneficial microbiota such as *Ruminococcus*, *Eubacterium*, and *Clostridium,* accompanied by an enrichment of pathobionts like *Enterococcus*. This microbial imbalance is thought to result from repeated dietary and antibiotic exposures that contribute to immune dysregulation, thereby facilitating the overgrowth of pathobionts and the development of SIBO, in contrast to the microbiome observed in healthy individuals [16].

#### 2.3.6. Predisposing Medications

Children with SBS often require complex and prolonged pharmacologic regimens to manage symptoms and support quality of life; however, these therapies may be associated with unintended downstream effects. Proton pump inhibitors have been postulated to play a role in predisposition to SIBO, given their alteration of gastric pH and disruption of acidic protective factors, while also being shown to disrupt the gut microbiome [1,28]. Some studies have demonstrated increased rates of SIBO on proton pump inhibitors (PPI) when using aspirates as diagnostic tools, but other studies using breath testing have not seen a significant difference between those on PPI and controls [3,10,20]. Other medications that can play a role are anti-diarrheal medications, which act to slow intestinal transit and peristalsis, decreasing mobilization of intestinal bacteria and increasing the risk of SIBO [29,30].

## 3. Prevalence

The prevalence of SIBO varies considerably depending on the clinical context, underlying comorbidities, and diagnostic method employed. Breath testing studies have shown that 10% to 60% of children with chronic functional GI symptoms, such as recurrent abdominal pain, bloating, and altered stool pattern, test positive for SIBO [1,3,31,32,33,34]. One study reported a 65% positivity rate among children meeting Rome III criteria for IBS, compared to only 7% in healthy pediatric controls [35]. Parenteral nutrition also increases risk, with close to 70% of patients presenting with SIBO in a retrospective study [12].

In pediatric populations, children with SBS have the highest documented prevalence of SIBO. Depending on the diagnostic method used, reported prevalence in this population ranges from 40% to 80% [1,2,3,4,9,11,26,36]. A retrospective pediatric cohort study demonstrates a 35% prevalence in SBS patients, while another prospective cohort study demonstrated a higher prevalence, finding that 64% of SBS patients exhibited clinical or biochemical evidence suggestive of SIBO [9,26]. Referencing the increased risk of SIBO while on PN, a multicenter review of pediatric intestinal rehabilitation programs across North America indicated that over 70% of children with SBS requiring long-term parenteral nutrition experienced at least one suspected or confirmed episode of SIBO [4]. Similarly, an additional retrospective study found a 6-fold increase in SIBO incidence among SBS patients receiving PN compared to those not receiving PN [36]. This data supports previous findings that the combination of anatomical disruption, PN dependence, and impaired motility in SBS increases the risk of bacterial overgrowth and recurrence rates [23].

Notably, the prevalence of SIBO in SBS is underestimated. Many children exhibit subclinical or intermittent symptoms, and commonly used breath tests may yield false negatives in patients with altered anatomy or rapid transit. Moreover, symptom recurrence is common; one study reported that up to 76% of symptom flares in SBS were attributed to SIBO [11]. In clinical practice, the high rate of recurrence often necessitates repeated courses of antibiotics, antimicrobial rotation, or adjunctive therapies such as probiotics or elemental diets.

## 4. Clinical Manifestations

**Figure 3:**
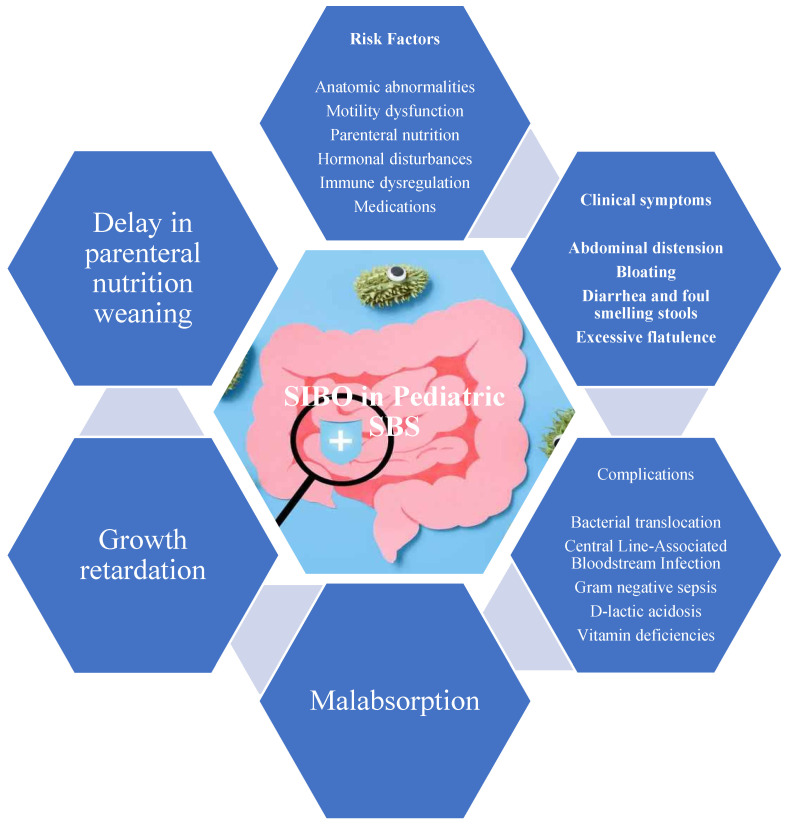
Clinical Characteristics of Small Intestinal Bacterial Overgrowth in Children with Short Bowel Syndrome.

The clinical presentation of SIBO in pediatric populations is diverse and often nonspecific, which complicates both diagnosis and clinical suspicion (Figure 3). The primary clinical manifestations of SIBO include gastrointestinal symptoms related to bacterial fermentation of unabsorbed carbohydrates, direct mucosal injury, and nutrient malabsorption [23,37]. Abdominal distension or bloating is among the most commonly reported symptoms, attributed to the production of hydrogen, methane, and carbon dioxide by proliferating microbial populations. Accumulation of these gases in the small intestine leads to bloating, discomfort, and altered bowel motility [3].

Diarrhea is another prominent and often early symptom of SIBO, which occurs due to multifactorial mechanisms. Bacterial deconjugation of bile acids impairs fat emulsification, leading to steatorrhea and osmotic diarrhea [36,38]. Inflammation caused by bacterial toxins and mucosal damage further impairs absorptive capacity, contributing to the diarrhea seen in SIBO.

Additionally, flatulence, a consequence of microbial fermentation, is commonly reported alongside bloating and diarrhea. Children may also experience cramping or generalized abdominal discomfort, although these symptoms are nonspecific and are often misattributed to functional gastrointestinal disorders or dietary intolerances [33]. Vomiting and nausea, although less frequently reported than diarrhea and bloating, may occur in younger children and could reflect upstream motility disturbances or bacterial toxin production [11]. These symptoms may be under-recognized in nonverbal children or those with neurodevelopmental disabilities, in whom discomfort is expressed atypically. Caregivers may report irritability, disrupted sleep, or feeding aversion, which can be mistakenly interpreted as behavioral issues rather than gastrointestinal pathology [33]. Less commonly, children with SIBO may present with malaise, fatigue, and decreased appetite.

In children with SBS, the symptom profile of SIBO is shaped by the anatomical and functional consequences of intestinal resection. Bloating is particularly prominent due to delayed transit and increased availability of fermentable substrates in the remaining bowel. These conditions favor extensive gas production and result in visible abdominal distension. Diarrhea, reported in up to 76% of episodes of suspected SIBO in SBS patients, is another prominent symptom noted in a single-center study [11]. Interestingly, some children with SBS report fewer overt symptoms than healthy controls with SIBO, due to the overlap between SIBO symptoms and baseline features of SBS [36,39]. Failure to thrive and growth retardation are significant concerns in SBS patients, as chronic diarrhea, inflammation, and nutrient malabsorption can lead to caloric deficiencies and malnutrition. The presence of SIBO can intensify malabsorption, hinder weaning from parenteral nutrition, and delay intestinal adaptation. Deficiencies in micronutrients, particularly vitamin B12, folate, iron, and fat-soluble vitamins (A, D, E, and K), are common due to bacterial competition, enzymatic destruction, and impaired mucosal uptake [3]. These deficiencies can lead to significant growth issues and nutritional complications.

In rare cases, severe bacterial overgrowth can lead to D-lactic acidosis, a condition particularly concerning in children with SBS. D-lactic acidosis results from the fermentation of carbohydrates by lactic acid-producing bacteria, leading to neurological symptoms such as confusion, ataxia, and lethargy [40]. Although rare, clinical suspicion of D-lactic acidosis has been reported in 8% of SIBO episodes in pediatric SBS patients, underscoring the importance of considering metabolic disturbances as part of the SIBO spectrum, especially in children with high-carbohydrate enteral feeds or abrupt dietary changes [6,40].

## 5. Diagnostic Approach

Diagnosing SIBO in pediatric populations presents unique challenges due to both the inherent limitations of existing diagnostic tools and the nonspecific nature of its clinical manifestations. While accurate identification of SIBO is essential to guide therapy and monitor recurrence, clinicians must often navigate a diagnostic landscape that is neither standardized nor uniformly validated in children. This diagnostic complexity is further amplified in patients with SBS, whose underlying anatomical and physiological alterations may confound test results or obscure symptomatology. Thus, a nuanced understanding of the available diagnostic modalities, their respective advantages and limitations, and the contextual variables in SBS is critical for effective evaluation of SIBO in pediatric care.

### 5.1. Endoscopic Sampling

#### 5.1.1. Small Intestinal Aspiration

The quantitative culture of jejunal or duodenal aspirates remains the most direct diagnostic method, but its utility is limited [13,31,41,42]. This procedure involves endoscopic or fluoroscopic-guided collection of small intestinal fluid, which is then cultured to determine CFU/mL. A diagnostic threshold of ≥10^3^ CFU/mL is commonly applied, though some guidelines recommend a higher cutoff of ≥10^5^ CFU/mL depending on the patient population and clinical context [1,3]. The advantage of this method lies in its ability to provide a direct microbial assessment and differentiate between bacterial species, which may theoretically inform antibiotic selection.

However, multiple limitations restrict its clinical use. The test is invasive, costly, and not widely available outside of specialized centers, particularly in pediatric practice, where sedation requirements add further risk [42]. Contamination with oropharyngeal microbiota during intubation may yield false positives, while the patchy distribution of bacterial overgrowth and failure to sample mid- or distal small bowel regions can result in false negatives [13,31,41,42]. Additionally, only about 30% of intestinal microbes are culturable, meaning aspirate cultures may substantially underestimate the true bacterial burden [42]. These factors, combined with logistical complexity and limited reproducibility, underscore why jejunal aspiration is best regarded as a useful but impractical research tool rather than a universally applicable gold standard in pediatric clinical settings [13,42].

#### 5.1.2. Brush Swabs and Mucosal Biopsies

More recent literature has demonstrated that additional endoscopic sampling methods may be consistent with aspirates. In a prospective cohort study, samples obtained from 44 children with SBS demonstrated no significant difference in SIBO diagnosis from different endoscopic sampling methods (intraluminal aspirate, epithelial brush swabs, and mucosal biopsies) [10]. These findings demonstrate that culture-based diagnosis of SIBO using brush swabs or mucosal biopsies may serve as viable alternatives to small bowel aspirates.

### 5.2. Breath Testing

**Figure 4:**
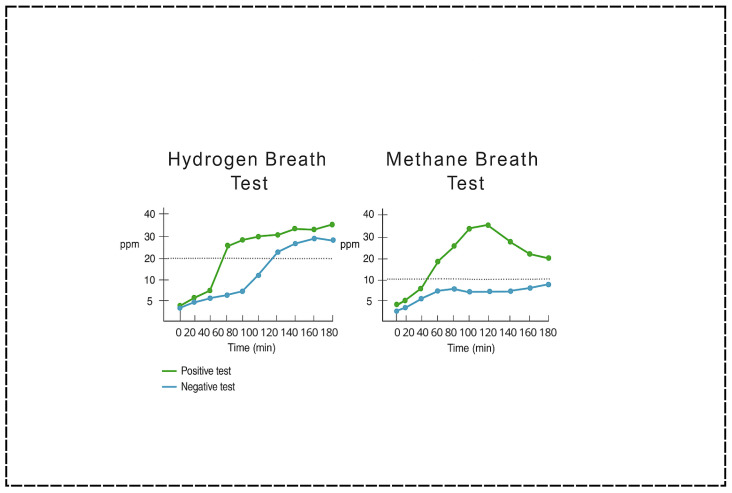
Representative breath test patterns illustrating hydrogen and methane profiles commonly observed in children with Small Intestinal Bacterial Overgrowth. This schematic figure was generated from sample breath test graphs obtained at our institution and is intended for illustrative purposes only and does not represent patient data.

Due to its non-invasive approach, breath testing has become the most widely utilized method for diagnosing SIBO in children [31]. Breath tests measure the concentration of hydrogen (H_2_) and methane (CH_4_) gases in exhaled air following the ingestion of a carbohydrate substrate—commonly glucose or lactulose (Figure 4). These gases are produced by intestinal microbes during fermentation of the administered carbohydrate and subsequently absorbed into the bloodstream and exhaled via the lungs [43]. An early rise in exhaled hydrogen (typically >20 parts per million above baseline within 90 min) is considered indicative of SIBO, as it suggests microbial activity in the small intestine rather than the colon [1,3]. Methane measurements may also be included to detect intestinal methanogen overgrowth (IMO), which is often associated with constipation-predominant symptoms and linked to the presence of *Methanobrevibacter smithii* [1].

#### 5.2.1. Glucose vs. Lactulose

Glucose hydrogen breath testing (GHBT) is often favored for its higher specificity. Glucose is rapidly absorbed in the proximal small intestine, and any hydrogen production more accurately reflects proximal small bowel bacterial activity [3]. In contrast, the lactulose hydrogen breath test (LHBT) is considered more sensitive because of its poor absorption profile, which allows it to reach more distal regions of the small intestine [11].

#### 5.2.2. Limitations of Breath Tests

While breath tests are attractive for their non-invasiveness and safety profile, especially in pediatric populations, they are not without significant limitations. Inconsistencies in test protocols—including variations in substrate type, dosing, interpretation criteria, and timing—have contributed to poor standardization [43,44]. A North American consensus report attempted to provide standardized guidelines for adults, but pediatric-specific protocols remain lacking [45]. Another critical issue is that there is no true gold standard against which breath testing can be validated. Jejunal aspiration, often considered the reference standard, presents limitations due to contamination risks, potential for sampling error, and a relatively low diagnostic yield [46]. Prior studies have also demonstrated that LHBT may not normalize after treatment despite clinical improvement, further complicating its role in longitudinal monitoring [28].

Test performance is highly dependent on strict patient adherence to preparatory protocols, such as withholding antibiotics for at least four weeks before testing, discontinuing promotility agents and laxatives, and following a prescribed dietary regimen [46]. Failure to comply with these restrictions can substantially reduce accuracy. Even under optimal conditions, the sensitivity and specificity of breath tests are moderate at best, ranging from 60 to 70% and 40 to 80%, respectively, depending on substrate choice and clinical context [31]. Younger children may also face practical challenges with breath collection due to limited respiratory coordination or cooperation, particularly under the age of five years.

The diagnostic reliability of breath testing may be further compromised by the altered anatomy and physiology of patients with SBS. Shortened intestinal length, absent ileocecal valves, and the presence of blind loops or fistulas can all alter substrate transit and microbial fermentation, leading to atypical gas production curves. For GHBT, specificity is higher, but sensitivity is limited: because glucose is absorbed almost entirely in the proximal small intestine, distal SIBO (e.g., in the ileum) may go undetected [41,46]. This limitation is particularly relevant in SBS patients, whose rapid transit may cause premature glucose absorption before it reaches distal sites of overgrowth. Conversely, LHBT is more sensitive but prone to false positives in the setting of rapid transit, also common in SBS [11].

### 5.3. Adjunctive Testing

#### 5.3.1. Quantitative PCR and Metagenome Sequencing

Emerging molecular methods include quantitative PCR (qPCR) and next-generation sequencing (NGS) to assess microbial DNA from stool or aspirate samples [44]. These approaches provide insights into microbial diversity and abundance, which may help differentiate between healthy and dysbiotic states. Metagenomic sequencing in SIBO and non-SIBO aspirates, swab brushings, and mucosal biopsies has demonstrated evidence of dysbiosis between groups, but not yet able to be used as a diagnostic tool, as colony numbers and variety are not consistent [10]. While promising, such methods require further validation in children with SBS and are not yet standardized for routine clinical use.

#### 5.3.2. Biomarkers

Biomarkers of intestinal inflammation and permeability have also been proposed as adjunctive tools in the diagnostic algorithm for SIBO. For instance, fecal calprotectin and serum zonulin levels may offer indirect evidence of mucosal dysfunction associated with microbial overgrowth [37]. However, these markers are nonspecific and cannot differentiate SIBO from other inflammatory or infectious enteropathies, limiting their standalone utility.

## 6. Management Strategies

**Figure 5:**
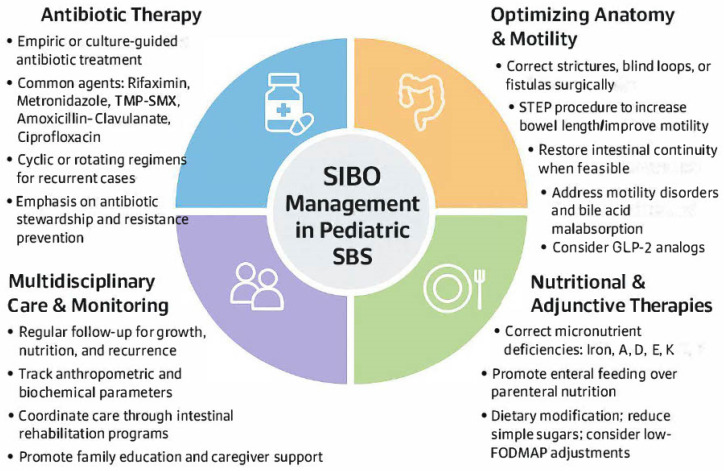
Overview of Management Strategies for Small Intestinal Bacterial Overgrowth in Children with Short Bowel Syndrome.

The treatment of SIBO in children with SBS presents a complex clinical challenge that necessitates a multifaceted and individualized approach. Because SIBO is not a primary disease but rather a secondary manifestation of underlying anatomical, motility-related, or immunological dysfunction, successful management hinges not only on antimicrobial therapy but also on addressing precipitating factors, supporting nutritional status, and preventing recurrence (Figure 5). Therapeutic strategies must be adapted to account for developmental physiology, antibiotic tolerability, and the inherent vulnerabilities of a surgically altered intestine.

### 6.1. Antibiotic Therapy

Some clinicians may adopt an empirical therapeutic trial as both a diagnostic and therapeutic tool in suspected cases of SIBO [45]. In this strategy, a short course of broad-spectrum antibiotics (e.g., rifaximin or metronidazole) is administered, and symptom resolution is used as indirect evidence of bacterial overgrowth [47]. While this approach avoids the pitfalls of unreliable testing, it also risks unnecessary antibiotic exposure and fails to provide definitive evidence of SIBO. In children with SBS, who often receive multiple courses of antibiotics for other complications, antibiotic stewardship is especially critical, and empirical therapy should be employed judiciously.

#### 6.1.1. Rifaximin

The mainstay of SIBO treatment is reducing bacterial overgrowth through antibiotics. Rifaximin, a non-systemically absorbed rifamycin derivative, is the most studied and frequently recommended agent. It offers broad-spectrum coverage against aerobic and anaerobic Gram-positive and Gram-negative bacteria, and its minimal systemic absorption reduces the risk of systemic side effects and antimicrobial resistance [28,48]. Data on the efficacy of rifaximin in pediatric patients with SIBO are limited. However, clinical experience and findings from small cohort studies suggest that rifaximin is generally well tolerated and effective in children, including those with functional abdominal pain and suspected SIBO [31]. Rifaximin’s lack of systemic absorption is particularly advantageous in the pediatric SBS population, given the frequency of repeated antibiotic exposures and the desire to minimize systemic antibiotic pressure [31].

#### 6.1.2. Alternative Microbial Coverage

Other antibiotics commonly used in pediatric SIBO include metronidazole, amoxicillin-clavulanate, ciprofloxacin, and trimethoprim-sulfamethoxazole (TMP-SMX) [49]. Selection is typically guided by availability, prior microbial exposure, and clinical history. In a retrospective cohort of children with SBS, metronidazole and TMP-SMX were the most frequently prescribed, with symptom resolution achieved in 56% of episodes following a single antibiotic course [11]. However, 44% required multiple regimens, reflecting the high rate of recurrence in this population. Metronidazole may be particularly effective in settings of blind loop syndrome, where systemic antibiotic absorption offers therapeutic advantages [50].

Although empiric antibiotics are effective, nearly 30% of patients experience persistent symptoms and recurrent SIBO, necessitating cyclic antibiotic regimens. This highlights the importance of individualized and sometimes prolonged treatment plans for children with SBS [51]. Evaluation of children with SIBO across diverse underlying pathologies revealed no significant differences in treatment response among metronidazole, rifaximin, and other commonly used antibiotics, suggesting comparable efficacy irrespective of the specific antimicrobial agent selected [51].

#### 6.1.3. Strategic Antimicrobial Cycling

The concept of cyclic or rotating antibiotic regimens has gained traction in managing recurrent or refractory SIBO, particularly in high-risk populations like those with SBS [48]. This strategy involves alternating antibiotics at defined intervals to prevent the development of resistance and minimize the disruption of commensal microbiota. However, pediatric data are lacking, and concerns about cumulative antibiotic exposure and gut microbiota disruption must be weighed carefully. In children with SBS, baseline microbial diversity is often compromised due to surgical resection and parenteral nutrition. Consequently, prudent antibiotic use is essential, as prolonged administration may lead to adverse outcomes, including antimicrobial resistance, reduced commensal microbial diversity, and the development of pathobionts [52]. In children with SBS who frequently require central venous access, chronic dysbiosis may predispose to gut-derived bloodstream infections and catheter-related sepsis. These risks underscore the importance of antibiotic stewardship and the integration of culture-directed therapy whenever feasible. While cyclical antibiotic administration is commonly employed for symptom control, a retrospective cohort study demonstrated that targeted therapy guided by endoscopic duodenal aspirate cultures significantly reduced emesis, feeding intolerance, and abnormal stool patterns, underscoring the value of quantitative analysis in patients unresponsive to empirical treatment [30].

### 6.2. Optimizing Anatomic Dysfunction

In addition to antimicrobial therapy, addressing anatomical and functional abnormalities that predispose children to SIBO is a critical component of long-term management. In the SBS population, stagnant segments of bowel, such as blind loops, strictures, or enteric fistulas, can contribute to bacterial pooling and overgrowth. Surgical correction of these issues, including resection of diseased segments, stoma closure, and restoration of bowel continuity, has been associated with reductions in SIBO incidence and improved nutrient absorption [11]. The serial transverse enteroplasty (STEP) procedure, which increases intestinal length and improves motility, has demonstrated success in reducing parenteral nutrition dependence and SIBO symptoms in select pediatric cases [53,54]. In patients with blind loop syndrome, surgical revision may be necessary to re-establish effective flow and reduce bacterial stasis [5]. These interventions illustrate the essential role of individualized surgical strategies in mitigating SIBO and enhancing intestinal adaptation in children with complex anatomical configurations.

### 6.3. Monitoring Nutrition and Growth

#### 6.3.1. Addressing Nutritional Deficiencies

Nutritional management represents another cornerstone of care in this population. Malabsorption of macronutrients and micronutrients—exacerbated by bacterial overgrowth—can lead to protein-energy malnutrition, growth failure, and multiple vitamin and mineral deficiencies [1,31]. Fat malabsorption due to bacterial deconjugation of bile acids can result in significant losses of vitamins A, D, E, and K, while bacterial competition for vitamin B12 and iron contributes to anemia and neurologic dysfunction [1,55]. Consequently, close monitoring and aggressive supplementation of at-risk nutrients—including B12, folate, iron, zinc, selenium, and fat-soluble vitamins—are essential. Serum albumin and prealbumin levels can help evaluate overall nutritional status, and enteral feeding should be encouraged to support mucosal health and intestinal adaptation, though formulations may need to be adjusted to reduce poorly absorbed carbohydrates that worsen fermentation-related symptoms [55].

#### 6.3.2. Probiotics and Dietary Strategies

Probiotics and dietary modifications have also been proposed as adjunctive therapies in the management of SIBO, although their roles in pediatric SBS remain incompletely understood. Some studies suggest that probiotic strains such as *Lactobacillus rhamnosus* GG and *Saccharomyces boulardii* may help restore microbial balance through barrier-enhancing and immunomodulating qualities, overall reducing SIBO-related symptoms [56,57]. *Lactobacillus rhamnosus* has been shown to displace pathogenic bacteria via inhibition of adhesion proteins, and *Bifidobacterium* can produce IgA-specific antimicrobial proteins that inhibit *E. coli* and *Klebsiella* [6,58]. Specified use of probiotics in SIBO was shown to improve rates of decontamination in the treatment arm by reducing H_2_ production and overall pain. However, there was no meaningful change in the overall prevention of SIBO while on probiotics [59]. It is important to note that clinical trials involving children are limited, and in immunocompromised or medically fragile patients, including those with SBS and central venous catheters, probiotics may pose a risk of developing fungemia or bacteremia. Thus, their use should be approached with caution and reserved for selected cases where benefits are likely to outweigh risks.

Dietary strategies aimed at reducing fermentable substrates for intestinal bacteria have gained attention in the management of SIBO [8]. Evidence supporting the efficacy of the low FODMAP (Fermentable Oligosaccharides, Disaccharides, Monosaccharides, and Polyols) diet in children with SBS and SIBO remains limited [1,3]. Moreover, in children with SBS, dietary restrictions must be balanced against the need for adequate caloric and nutrient intake. Simple measures such as reducing simple sugars and lactose in the diet may be helpful in some cases, particularly when guided by symptoms and tolerance.

In SBS, dietary management is further complicated by the frequent reliance on PN. Because PN is associated with an increased risk of SIBO, optimizing enteral feeding whenever possible remains a critical goal [60,61,62]. In a retrospective cohort study of 16 pediatric patients with SBS receiving home PN, SIBO was significantly associated with failure to achieve enteral autonomy. Among those who remained dependent on PN, 62.5% were found to have concomitant SIBO. Notably, none of the children diagnosed with both SBS and SIBO attained bowel autonomy [11]. These findings suggest that SIBO may represent a key barrier to successful weaning from PN, underscoring the clinical importance of identifying and managing SIBO to support the transition to enteral nutrition.

#### 6.3.3. Ensuring Adequate Growth

From a growth perspective, anthropometric measurements should be tracked longitudinally using age-appropriate growth charts, with careful attention to weight-for-age, height-for-age, and body mass index. Declining z-scores or crossing of growth percentiles may signal worsening malabsorption or recurrent SIBO [63]. In cases of prolonged nutritional compromise, additional assessments such as bone age radiographs or dual-energy X-ray absorptiometry (DEXA) scans may be warranted to evaluate growth potential and bone health. Together, this integrated approach to laboratory and nutritional monitoring is critical to mitigating the complications of SIBO in SBS and supporting long-term health and development in affected children.

### 6.4. GLP-2 Analogs

GLP-2 analogs, such as teduglutide, offer promise in supporting mucosal integrity and enhancing adaptation, which may indirectly reduce the risk or severity of SIBO [15,64]. GLP-2 stimulates crypt cell proliferation, increases villus height, and enhances barrier function—all properties that may be beneficial in children with SBS and chronic SIBO [15]. Preliminary data in limited pediatric series suggest that GLP-2 analogs may reduce dependence on parenteral nutrition and facilitate enteral autonomy, indirectly mitigating one of the major risk factors for SIBO [61,64,65].

### 6.5. Prioritizing Multidisciplinary Involvement

Finally, the multidisciplinary nature of SIBO management in children with SBS cannot be overstated. Effective treatment often requires coordination among pediatric gastroenterologists, surgeons, dietitians, infectious disease specialists, and nursing staff. Regular follow-up to assess growth, nutritional status, laboratory indices, and symptom recurrence is essential. Many institutions utilize intestinal rehabilitation programs to coordinate care for children with SBS, and these settings are ideal for comprehensive SIBO management as well. Despite advances in intestinal rehabilitation, significant heterogeneity exists among pediatric intestinal rehabilitation programs regarding SIBO diagnosis and treatment protocols. A recent international survey highlighted variation in the use of breath testing, antibiotic selection, and empiric therapy thresholds, reflecting the absence of standardized pediatric guidelines [66]. This variability underscores the need for a consensus-driven clinical algorithm that standardizes practice and strengthens the quality and comparability of research in this complex population.

## 7. Limitations

This review has several limitations that should be considered when interpreting its findings. Most available studies investigating SIBO in children with SBS are retrospective and involve small, heterogeneous cohorts, limiting statistical power and generalizability. Differences in patient age, intestinal anatomy, and degree of PN dependence further complicate comparisons across studies. Diagnostic heterogeneity remains a major methodological constraint: while some studies rely on hydrogen or methane breath testing, others use jejunal aspirates or clinical criteria, each with varying sensitivity and specificity in pediatric SBS. The lack of standardized cutoff values and inconsistent sampling techniques introduces potential bias and hinders meta-analytic synthesis.

Additionally, many reports originate from single-center tertiary care programs, which may not reflect outcomes in community or resource-limited settings. Publication bias toward studies with positive results may also overestimate the true prevalence and impact of SIBO in this population. Finally, few studies incorporate microbiome sequencing or longitudinal assessment, restricting insight into causal relationships between dysbiosis, intestinal adaptation, and clinical outcomes. Future multicenter, prospective studies employing standardized diagnostic methods are essential to validate current observations and guide evidence-based management of pediatric SIBO in SBS.

## 8. Future Directions

SIBO represents a clinically significant and recurring complication in pediatric patients with SBS. Rather than a discrete infection, SIBO reflects broader pathophysiological disturbances in motility, mucosal immunity, and intestinal anatomy. In children reliant on PN or undergoing intestinal rehabilitation, SIBO exacerbates malabsorption, complicates feeding regimens, and delays progress toward enteral autonomy.

Diagnostic options for SIBO in children with SBS remain limited. Breath testing is widely used due to its safety and accessibility, but it is often unreliable in children with altered intestinal transit or anatomy. Gold-standard jejunal aspiration presents limitations, and standardization of testing protocols remains elusive. Future research must focus on validating pediatric-specific diagnostic methods, potentially incorporating non-invasive tools such as microbiome profiling and metabolomic biomarkers.

Current treatment strategies rely predominantly on antibiotics, with agents such as rifaximin and metronidazole demonstrating clinical benefit. However, recurrence is common, and repeated exposure poses risks of antimicrobial resistance and further disruption of the intestinal microbiota. In SBS patients, whose microbiota are already disrupted, this risk is even greater. Studies are needed to define optimal treatment duration, cycling protocols, and the long-term consequences of chronic antibiotic use in children.

Adjunctive approaches—including dietary modification, probiotics, and enteral formula adjustments—warrant further investigation. Evidence supporting the efficacy of the low-FODMAP diet and specific probiotic strains remains limited. Additionally, safety concerns regarding probiotic use in immunocompromised or catheter-dependent children necessitate careful patient selection. Beyond these measures, future controlled trials are necessary to explore the efficacy of GLP-2 therapy in preventing and managing SIBO in pediatric SBS patients.

## 9. Conclusions

In summary, pediatric SIBO in patients with SBS demands nuanced, personalized management. As our understanding of the microbiome, motility, and host-immune interactions deepens, future care will increasingly rely on targeted therapies and predictive diagnostics. Ongoing research, guideline development, and multidisciplinary coordination will be key to improving outcomes and quality of life for this vulnerable patient population.

## Figures and Tables

**Table 1:** Factors Contributing to Small Intestinal Bacterial Overgrowth in Pediatric Short Bowel Syndrome.

Anatomical alteration
Surgical blind loops Strictures Resection of the ileocecal valve Gastrocolic or jejunocolic fistula Small intestinal diverticulosis
Intestinal motility disturbance
Intestinal dysmotility, gastroparesis Associated inflammatory conditions like Crohn’s disease Hypochlorhydria or achlorhydria Reduction in gut-associated lymphoid tissue after resection Autonomic neuropathy
Multifactorial
Parenteral nutrition Medications—acid suppression therapies, opiate use Immunodeficiency states Chronic pancreatitis Liver disease

## Data Availability

Not applicable.
